# Arteannuin B Inhibits NSCLC Cells via Regulating miR‐194‐3p/ *CLDN2* Axis

**DOI:** 10.1002/cam4.71796

**Published:** 2026-04-14

**Authors:** Ting‐Sha He, Rong‐Hui Chen, Jing Feng, Qiang Zhang, Xin‐Ling Li, Jia‐Hui Han, Tian‐Ze Chen, Rong‐Min Yu, Li‐Yan Song, Wei‐Juan Huang

**Affiliations:** ^1^ Department of Pharmacology, College of Pharmacy Jinan University Guangzhou China; ^2^ Biotechnological Institute of Chinese Materia Medica Jinan University Guangzhou China; ^3^ Department of Chinese Materia Medica, College of Pharmacy Jinan University Guangzhou China

**Keywords:** Arteannuin B, chemoresistance, *CLDN2*, miR‐194‐3p, NSCLC

## Abstract

Arteannuin B (Art B), a sesquiterpene lactone from *Artemisia annua* , combats non‐small cell lung cancer (NSCLC) chemoresistance by activating a novel miR‐194‐3p/CLDN2 axis, as identified here through integrated transcriptomic and functional analyses. Here, we combined transcriptomic profiling with functional validation to identify claudin‐2 (CLDN2) as a critical mediator of Art B's anticancer effects in NSCLC. *CLDN2*, significantly upregulated in NSCLC tissues versus paired normal tissues, promoted tumor cell proliferation and cisplatin resistance by upregulating multidrug resistance‐associated protein 2 (MRP2). Mechanistically, miR‐194‐3p directly binds to two conserved sites (nt 358–365 and 1232–1238) within the *CLDN2* 3′ UTR, suppressing its expression via mRNA degradation and translational inhibition, thereby attenuating proliferation and resensitizing cells to cisplatin. Importantly, Art B exerted antitumor effects by upregulating *miR‐194‐3p*, which subsequently inhibited *CLDN2*. This study not only elucidates a previously unrecognized mechanism for overcoming chemoresistance but also nominates CLDN2 as a prognostic biomarker, offering a promising therapeutic strategy that could benefit NSCLC patients facing treatment failure.

## Graphical abstract

Arteannuin B (Art B), a sesquiterpene lactone from *Artemisia annua*, upregulates *miR‐194‐3p*, which directly targets two conserved sites (nt 358–365 and 1232–1238) in the CLDN2 3′ UTR. This triggers mRNA decay and blocks translation, suppressing CLDN2 expression. Downregulation of CLDN2 inhibits MRP2‐mediated cisplatin (DDP) resistance in non‐small cell lung cancer (NSCLC). The right panel illustrates the oncogenic role of CLDN2 in promoting MRP2 expression and chemoresistance.

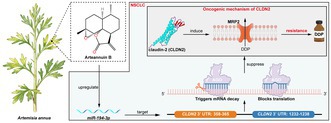

## 1 Introduction

Lung cancer remains the most lethal malignancy globally, with non‐small cell lung cancer (NSCLC) accounting for approximately 85% of cases and being characterized by aggressive progression, intrinsic therapeutic resistance and early metastatic dissemination [1, 2]. NSCLC is histologically classified into adenocarcinoma, squamous cell carcinoma and large cell carcinoma, each driven by distinct molecular alterations such as *EGFR* mutations and *ALK* rearrangements [3]. Despite advancements in targeted therapies and immunotherapies, a significant proportion of patients acquire resistance, which ultimately leads to treatment failure and poor prognosis [4]. This persistent challenge highlights the imperative to elucidate biomarkers and mechanisms governing NSCLC progression and therapeutic resistance.

Artemisinin derivatives, low‐toxicity sesquiterpene lactones isolated from *Artemisia annua* , are widely recognized for their antimalarial efficacy and exhibit broad therapeutic potential in oncology [5, 6, 7]. Beyond their antiparasitic applications, these compounds selectively impair cancer cell viability through mechanisms such as reactive oxygen species (ROS)‐mediated cytotoxicity, angiogenesis suppression via VEGF signaling pathway inhibition, and reversing multidrug resistance through inhibition of drug efflux transporters [8, 9, 10]. Among these derivatives, arteannuin B (Art B) has demonstrated anti‐inflammatory and anticancer efficacy in preclinical models, including osteoporosis and neuroinflammatory disorders [11, 12]. However, its molecular targets and functional roles in NSCLC, particularly in overcoming chemoresistance, remain uncharacterized, hindering its clinical translation due to undefined molecular targets [13, 14].

The claudin family, first identified in 1998, comprises tetraspan transmembrane proteins encoded by genes essential for maintaining epithelial barrier integrity and cell polarity [15, 16]. Claudin‐2 (CLDN2), significantly upregulated in NSCLC compared to adjacent normal tissues, has emerged as a pivotal oncogenic driver [17, 18, 19]. *CLDN2* promotes tumor progression through ligand‐independent activation of Epidermal Growth Factor Receptor (EGFR)/Extracellular Signal‐Regulated Kinase (ERK) signaling pathway, disruption of epithelial‐mesenchymal transition (EMT) dynamics via E‐cadherin downregulation, and enhancement of chemoresistance through *Sp1*‐mediated upregulation of multidrug resistance‐associated protein 2 (MRP2/ABCC2) [20, 21, 22, 23]. Notably, *CLDN2* knockdown restores chemosensitivity by increasing intracellular accumulation of platinum‐based agents, directly linking its expression to therapeutic failure [24]. Given its central role in driving proliferation and, most importantly, chemoresistance, CLDN2 represents a compelling therapeutic target for overcoming treatment failure in NSCLC.

Aberrantly expressed microRNAs (miRNAs) are increasingly implicated in NSCLC tumorigenesis and therapy resistance, with emerging roles in regulating claudin family members [25]. While miRNAs are known to regulate claudin family members in different malignancies, their roles in *CLDN2*‐driven NSCLC progression and chemoresistance remain unexplored [26]. This study aims to (1) elucidate *CLDN2*'s functional contributions to NSCLC progression, (2) identify upstream miRNA regulators of *CLDN2* and (3) evaluate the efficacy of Art B in suppressing *CLDN2*‐mediated chemoresistance. We hypothesize that the natural compound Arteannuin B (Art B) exerts its antitumor effects by modulating a specific miRNA to inhibit CLDN2, thereby suppressing proliferation and reversing cisplatin resistance in NSCLC.

## 2 Materials and Methods

### 2.1 Bioinformatic Analysis of *CLDN2* Expression

*CLDN2* expression profiles and associated clinical metadata (tumor/normal status, sex) for lung adenocarcinoma (LUAD) and squamous cell carcinoma (LUSC) cohorts were extracted from UCSC Xena‐processed TCGA data sets. Normalized RNA‐seq by expectation–maximization (RSEM) values (log_2_‐transformed) for *CLDN2* were stratified by tissue type (tumor vs. adjacent normal) and sex. Batch effects were mitigated using ComBat (sva R package, v3.48.0), and samples with low *CLDN2* expression (median transcripts per million (TPM) < 1) were excluded. Differential expression analysis between tumor and normal tissues was performed with limma (v3.56.2), incorporating sex as a covariate.

### 2.2 Cell Culture

Human NSCLC cell lines A549, HCC827, H1299, and H460 were acquired from the Cell Bank of the Chinese Academy of Sciences (Shanghai, China). Cells were propagated in RPMI 1640 medium supplemented with 10% fetal bovine serum (FBS) and maintained under standard culture conditions (37°C, 5% CO_2_, humidified atmosphere).

### 2.3 Plasmids and Transfection

*CLDN2*‐targeting siRNA and scrambled control siRNA were sourced from Sangon Biotech (Shanghai, China). *Hsa‐miR‐194‐3p* mimics, mimic negative controls (NC), inhibitors, and inhibitor NCs were acquired from RiboBio (Guangzhou, China). Plasmid transfections were performed using Lipofectamine 3000 (Thermo Fisher Scientific) according to the manufacturer's guidelines.

### 2.4 MTT Assay

Following transfection, NSCLC cells were plated in 96‐well plates (1 × 10^3^ cells/well) and allowed to adhere for 24 h in complete medium. DDP and Art B were diluted to target concentrations, applied to respective wells and incubated for 48 h. MTT solution (5%, 20 μL) was then introduced, followed by a 4‐h incubation. Formazan crystals were solubilized with dimethyl sulfoxide (DMSO; 200 μL/well) under light‐protected conditions, and plates were agitated for 10 min. Absorbance at 570 nm was measured using a microplate reader, and cell viability was quantified relative to untreated controls.

### 2.5 Colony Formation Assay

Transfected NSCLC cells were plated in 6‐well plates at a density of 1 × 10^3^ cells/well and maintained in culture medium for 14 days. Colonies were fixed with 4% paraformaldehyde (PFA; 30 min) and stained with 0.5% crystal violet (30 min, room temperature).

### 2.6 Flow Cytometry

Apoptosis was assessed using an Annexin V/PI detection kit (4A Biotech, Beijing) according to the manufacturer's protocol. Cells were detached with 0.25% EDTA‐free trypsin, washed twice with ice‐cold PBS and resuspended in binding buffer prior to staining. For intracellular DDP accumulation, cells underwent identical trypsinization and PBS washing steps, followed by analysis of DDP‐FITC fluorescence intensity. All samples were processed on a FACSCanto II flow cytometer.

### 2.7 Western Blot Analysis

Cells were lysed 48 h post‐transfection using RIPA buffer (Beyotime). Protein lysates were resolved on 10% SDS‐PAGE gels and transferred to nitrocellulose membranes (Millipore). Membranes were probed overnight at 4°C with primary antibodies: rabbit anti‐CLDN2 (Abcam), rabbit anti‐CTR1 (Abcam), rabbit anti‐MRP2 (Abcam), and mouse anti‐*β*‐actin (Cell Signaling Technology). After TBST washes, membranes were incubated for 1 h at room temperature with HRP‐conjugated secondary antibodies: goat anti‐rabbit IgG for CLDN2, CTR1, and MRP2 and goat anti‐mouse IgG for *β*‐actin. Signal detection was performed using a chemiluminescent substrate (ECL; Beyotime) following three 10‐min TBST washes.

### 2.8 Quantitative Real‐Time PCR (qRT‐PCR)

Total RNA was isolated from NSCLC cells using TRIzol reagent (Sangon Biotech, Shanghai, China) and reverse‐transcribed into cDNA with a RevertAid First Strand cDNA Synthesis Kit (Yeasen Biotech, Shanghai, China). qRT‐PCR was performed using SYBR Green Master Mix on a QuantStudio 6 Flex system (Thermo Fisher Scientific). Primer sequences for *miR‐194‐3p* and U6 (RiboBio, Guangzhou, China) are provided in Table S1. Relative gene expression was calculated via the 2^−ΔΔCt^ method, normalized to U6 (for miRNA) and *GAPDH* (for mRNA).

### 2.9 Tumor Xenograft Model

Male BALB/c nude mice (*n* = 32, 3–4 weeks old, specific pathogen‐free) were acquired from the Institute of Laboratory Animal Sciences (Chinese Academy of Medical Sciences) and acclimatized under controlled environmental conditions. A549 cells (2 × 10^6^ cells in 100 μL PBS) were subcutaneously injected into the left flank. Tumor volume was monitored twice weekly using calipers and calculated as V = length × width^2^ × 0.5. Drug administration commenced when tumors reached 70 mm^3^. After 28 days, mice were euthanized, and tumors were excised, weighed, and processed for downstream analysis.

### 2.10 Dual‐Luciferase Reporter Assay

Putative *miR‐194‐3p* binding sites within the *CLDN2* 3′ UTR were predicted using miRDB and TargetScan algorithms. Wild‐type *CLDN2* 3′ UTR sequences were cloned into pmirGLO vectors (Promega), while scrambled sequences served as controls. 293 T cells were co‐transfected with pmirGLO‐*CLDN2* 3′ UTR (or control vector), *miR‐194‐3p* mimic, or mimic negative control (NC) using Lipofectamine 3000. Luciferase activity was measured 48 h post‐transfection using a dual‐luciferase assay system (Yeasen Biotech), with Renilla luciferase normalized to firefly luciferase for each sample.

### 2.11 Statistical Analysis

All statistical analyses were conducted in R (v4.4.2). Continuous data are expressed as mean ± SD (*n* = 3). Differences between two groups were evaluated using unpaired two‐tailed Student's *t*‐tests. For multi‐group comparisons, two‐way ANOVA followed by Tukey's post hoc test was applied. A significance threshold of *p* < 0.05 was used for all analyses.

### 2.12 Data Visualization and Statistical Annotation

Data visualization was implemented using the ggplot2 package (v3.4.2) for layered plot construction. Color palettes were optimized for accessibility and perceptual uniformity via viridis (v0.6.2). Axes scaling and labeling were standardized using scales (v1.2.1). Statistical annotations (e.g., significance brackets, adjusted *p*‐values) were generated with ggpubr (v0.6.0) and validated through non‐parametric testing workflows in rstatix (v0.7.2).

## 3 Results

### 3.1 Cross‐Omics Profiling Identifies *CLDN2* Suppression by Art B Attenuates NSCLC Progression

**FIGURE 1:**
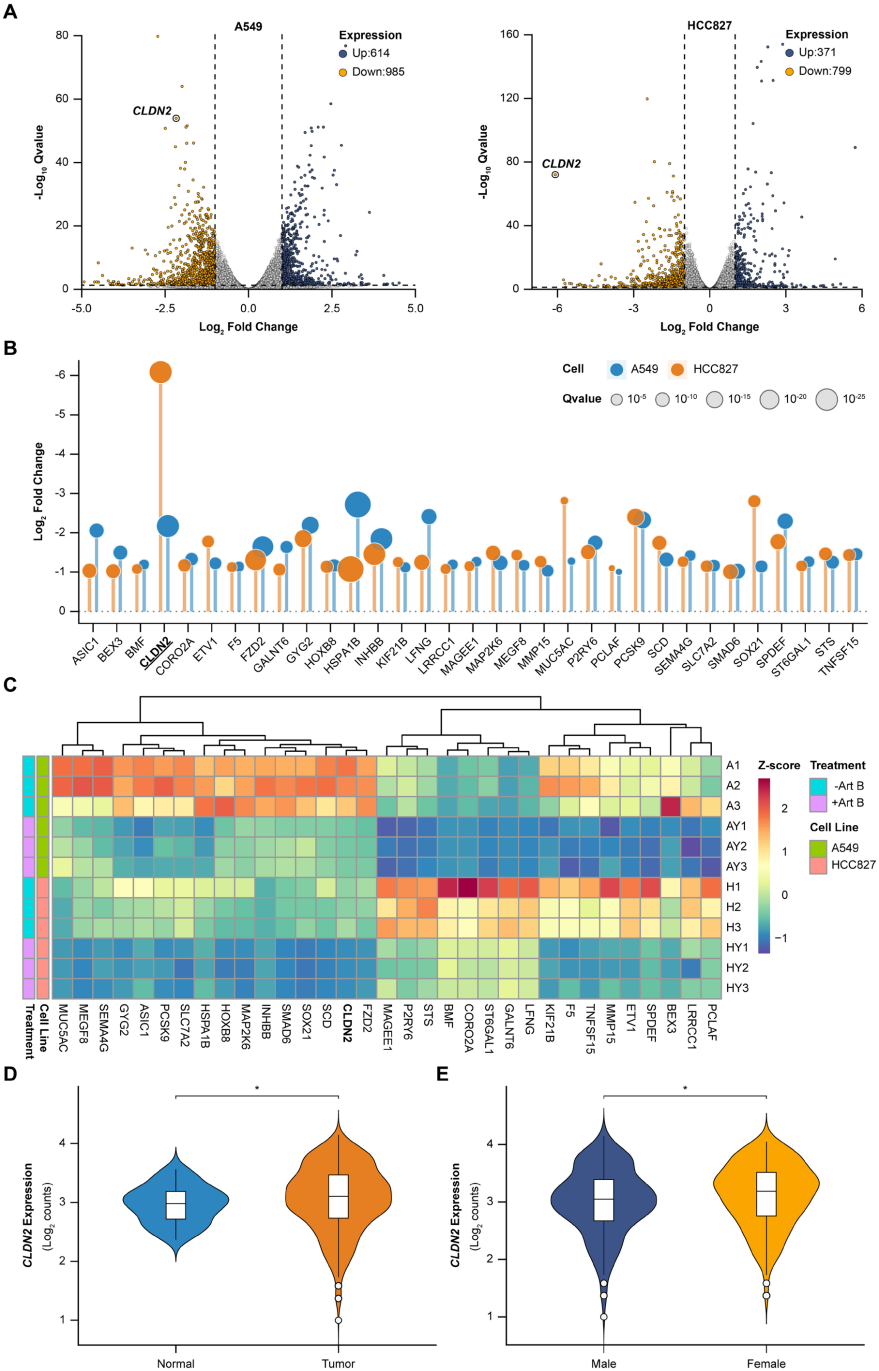
*CLDN2* expression modulation by Art B in NSCLC models and clinical cohorts. (A) Volcano plots of Art B‐treated A549 (left) and HCC827 (right) cells. Downregulated genes (orange), upregulated genes (dark blue). Cells were treated with 6.25 μM Art B (*n* = 3). Dashed lines indicate thresholds (|Log_2_ Fold Change| > 1, −Log_10_ Q > 1.3). (B) Ranked differential expression analysis of co‐downregulated genes in A549 and HCC827. A549 (blue), HCC827 (orange). Dot size represents statistical significance (*Q* value). (C) *Z*‐score heatmap of *CLDN2* expression across biological replicates (A1–A3: A549; H1–H3: HCC827). Heatmap depicts Z‐score normalized CLDN2 mRNA expression (*n* = 3). “AY” or “HY” denotes Art B‐treated samples compared to vehicle control (“A” or “H”). (D) *CLDN2* expression in LUAD/LUSC tumors (orange) versus normal tissues (blue). Data are derived from the TCGA database. Student's *t*‐test, *p < 0.05. (E) Sex‐stratified *CLDN2* expression in NSCLC patients (female: Orange; male: Blue). Analysis based on TCGA cohort. Student's *t*‐test, *p < 0.05.

To delineate the molecular targets of Art B, we performed transcriptomic profiling in NSCLC cell lines (A549, HCC827). RNA‐seq analysis of Art B‐treated A549 cells revealed 985 downregulated and 614 upregulated genes (Figure 1A). HCC827 cells showed a concordant suppression trend with 799 downregulated genes, though the magnitude of change was smaller than in A549 cells. Intersection analysis of co‐downregulated genes identified *CLDN2* as the most significantly suppressed target in A549 cells (Figure 1B), exceeding its attenuation in HCC827 and ranking it within the top 1% of shared pharmacodynamic responders.

*CLDN2* suppression displayed cell line‐specific dynamics, with pronounced knockdown efficiency in A549 cells compared to attenuated yet consistent downregulation in HCC827. Although inter‐sample variability persisted in HCC827 cohorts, replicate‐level analyses confirmed directional suppression aligned with negative *Z*‐score distributions (Figure 1C).

In clinical validation, TCGA analysis of LUAD/LUSC tissues confirmed elevated *CLDN2* expression in tumors compared to matched normal tissues (Figure 1D). Sex‐stratified analysis, informed by *CLDN2*'s X‐chromosome localization, revealed higher baseline expression in female NSCLC patients (Figure 1E), consistent with its pro‐tumorigenic function.

Collectively, our data identify CLDN2 as a key pharmacodynamic target of Art B in NSCLC, underscoring its promise as a therapeutic target given its elevated expression in tumors, particularly in females.

### 3.2 *CLDN2* Mediates Art B's Suppression of NSCLC Across Models

**FIGURE 2:**
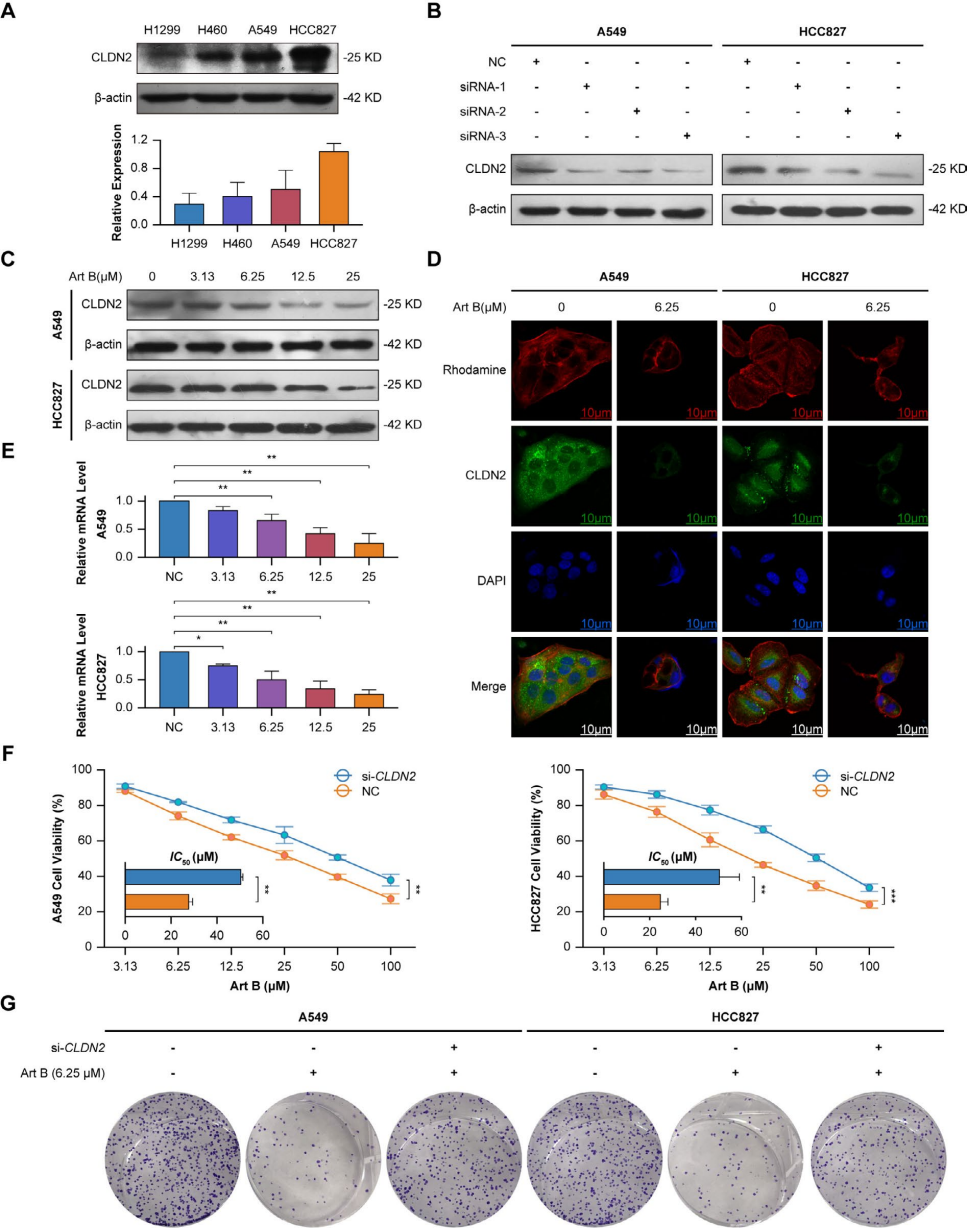
*CLDN2* expression modulation and functional analysis in NSCLC models. (A) Western blot analysis of *CLDN2* in H1299, H460, A549, and HCC827 cell lines (*n* = 3). (B) *CLDN2* protein levels in A549 and HCC827 following transfection with siRNA constructs (NC, siRNA‐1, siRNA‐2, siRNA‐3) (*n* = 3). (C) *CLDN2* suppression in A549 and HCC827 treated with increasing concentrations of Art B (0–25 μM) (*n* = 3). (D) Immunofluorescence imaging of *CLDN2* (green), Rhodamine (red), and DAPI (blue) in Art B‐treated cells (6.25 μM). (Scale bars: 10 μm, *n* = 3). (E) *CLDN2* mRNA levels in A549 and HCC827 post‐Art B treatment (0–25 μM). Data are expressed as mean ± SD (*n* = 3). Student's *t*‐test, *p < 0.05, **p < 0.01. (F) Cell viability curves of Left: A549 and Right: HCC827 under escalating Art B concentrations (3.13–100 μM). The histogram displays the *IC*_50_ values corresponding to each curve. Data are expressed as mean ± SD (*n* = 3). Cell viability curves using curves two‐way ANOVA, **p < 0.01, ***p < 0.001; histogram using Student's *t*‐test, **p < 0.01. (G) Colony formation assays in A549 and HCC827 under indicated treatments (*n* = 3).

Western blot analysis revealed elevated *CLDN2* protein expression in A549 and HCC827 cells compared to H1299 and H460 (Figure 2A). Through screening of siRNA constructs targeting *CLDN2*, siRNA‐3 demonstrated the highest knockdown efficiency, achieving near‐complete CLDN2 suppression in both A549 and HCC827 cells (Figure 2B). Based on this efficacy, *CLDN2*‐siRNA 3 (si‐*CLDN2*) was selected for subsequent experiments.

Art B treatment caused dose‐dependent *CLDN2* suppression. At 25 μM, *CLDN2* protein was nearly undetectable in A549 cells but retained residual expression in HCC827 cells, attributable to higher basal CLDN2 levels in the latter cell line (Figure 2C). Immunofluorescence imaging revealed marked reduction of *CLDN2* expression in Art B‐treated cells, concurrent with decreased cancer cell density, both inversely correlated to escalating drug concentrations (Figure 2D). Transcriptional suppression paralleled protein downregulation, with *CLDN2* mRNA levels decreasing significantly in response to escalating Art B doses (Figure 2E).

si‐*CLDN2* attenuated Art B's anti‐proliferative effects. In A549 cells, Art B's inhibitory potency was significantly reduced in si‐*CLDN2*‐treated groups, reflected by a substantial increase in *IC*_50_ (Figure 2F). Colony formation assays revealed partial restoration of clonogenic capacity in si‐*CLDN2*‐transfected HCC827 cells under Art B treatment (Figure 2G). These results establish *CLDN2* as a key mediator of Art B's anti‐NSCLC activity.

Our functional studies establish *CLDN2* as a central executor of Art B's action. Art B transcriptionally and translationally represses *CLDN2* in a dose‐dependent manner, while genetic silencing of *CLDN2* diminishes its therapeutic efficacy, confirming a direct mechanistic link between target suppression and anti‐tumor outcome.

### 3.3 *CLDN2* Depletion Potentiates Cisplatin Sensitivity in NSCLC

**FIGURE 3:**
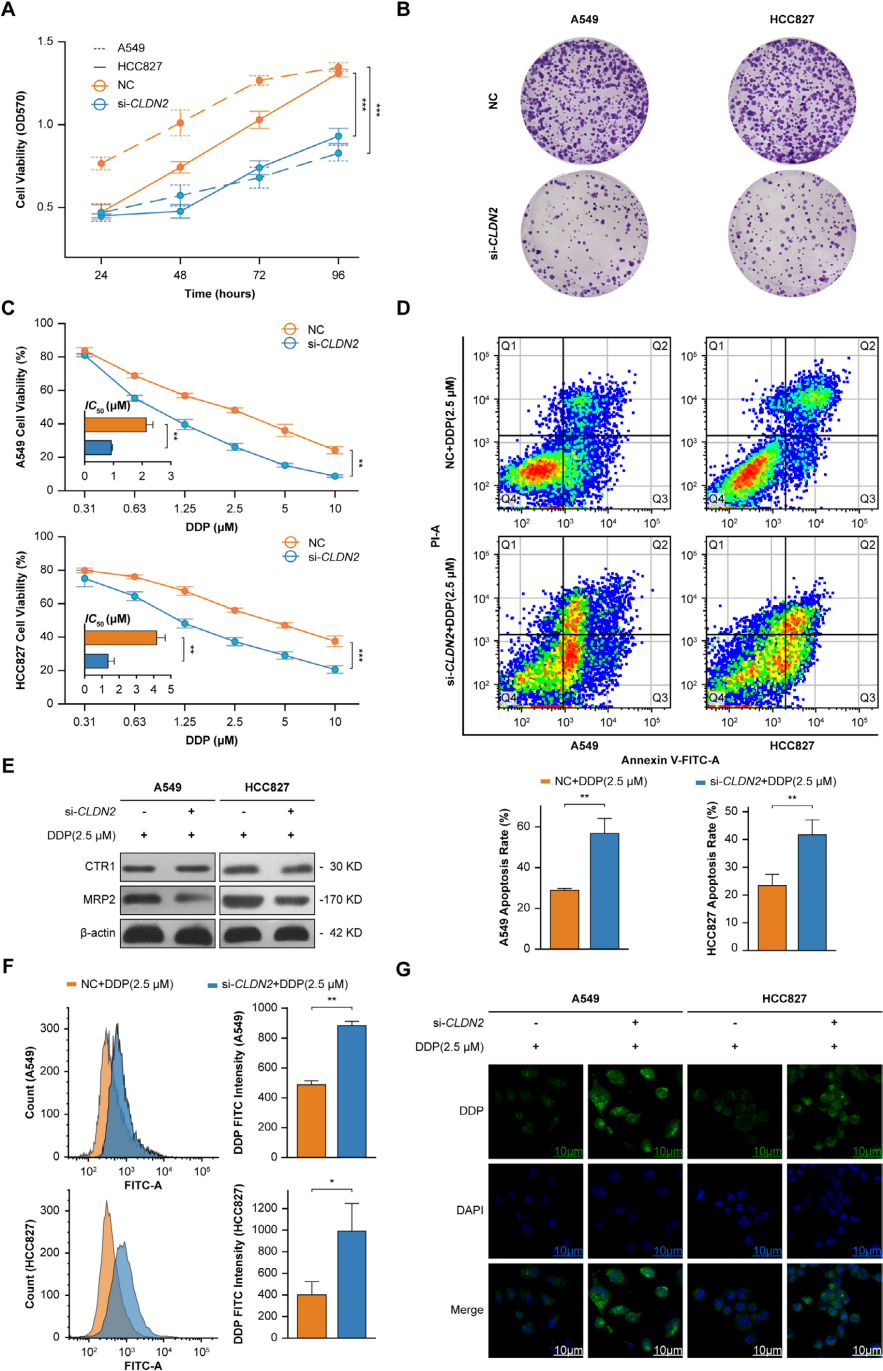
*CLDN2* depletion alters cisplatin (DDP) sensitivity and cellular phenotypes in NSCLC models. (A) Time‐dependent cell viability in A549 (dashed line) and HCC827 (solid line) cells treated with NC (orange) or si‐*CLDN2* (blue). Data are expressed as mean ± SD (*n* = 3). Two‐way ANOVA, ***p < 0.001. (B) Morphological changes in NC and si‐*CLDN2* groups (*n* = 3). (C) Top: A549 and Bottom: HCC827 viability under escalating DDP concentrations (0.31–10 μM). The histogram displays the *IC*_50_ values corresponding to each curve. Data are expressed as mean ± SD (*n* = 3). Cell viability curves using curves two‐way ANOVA, **p < 0.01, ***p < 0.001; histogram using Student's *t*‐test, **p < 0.01. (D) Annexin V/PI flow cytometry plots comparing NC + DDP (2.5 μM) and si‐*CLDN2* + DDP (2.5 μM) groups. Data are expressed as mean ± SD (*n* = 3). Student's *t*‐test, **p < 0.01. (E) MRP2 and CTR1 protein expression by Western blot (*n* = 3). (F) Left: Apoptotic cell quantification; Right: DDP fluorescence intensity in NC + DDP (2.5 μM) (orange) and si‐*CLDN2* + DDP (2.5 μM) (blue). Data are expressed as mean ± SD (*n* = 3). Student's *t*‐test, *p < 0.05, **p < 0.01. (G) Fluorescence microscopy of intracellular DDP distribution (green) co‐stained with DAPI (blue) in NC and si‐*CLDN2* groups (scale bars: 10 μm, *n* = 3).

*CLDN2* depletion induced sustained suppression of NSCLC cell proliferation. Compared to controls, si‐*CLDN2*‐treated A549 and HCC827 cells exhibited progressive viability loss and near‐complete elimination of clonogenic capacity (Figure 3A,B). Morphological analysis revealed marked cellular shrinkage and nuclear fragmentation, consistent with impaired proliferative potential (Figure 3B).

The combination of *CLDN2* knockdown with cisplatin (DDP) demonstrated synergistic cytotoxicity, as shown by dose‐dependent reductions in viability and a pronounced shift toward late‐stage apoptotic populations (Figure 3C,D). *CLDN2* depletion thus not only suppressed proliferation but also enhanced DDP sensitivity in NSCLCs. To elucidate the molecular basis of this chemosensitization, we evaluated two critical determinants of platinum pharmacokinetics: multidrug resistance‐associated protein 2 (MRP2), a drug efflux pump, and copper transporter 1 (CTR1), the primary platinum influx channel.

Mechanistic studies showed selective downregulation of MRP2 in *CLDN2*‐deficient cells, with CTR1 expression remaining unaffected (Figure 3E). Flow cytometry analysis revealed prolonged intracellular DDP retention in si‐*CLDN2*‐treated cells (Figure 3F), with spatially resolved fluorescence microscopy further confirming perinuclear drug aggregation—a spatial signature of efflux pathway disruption (Figure 3G). Therefore, we hypothesize that *CLDN2* depletion enhances cisplatin sensitivity in NSCLC by suppressing MRP2‐mediated drug efflux, leading to prolonged intracellular cisplatin retention and synergistic cytotoxicity through impaired proliferation and enhanced apoptosis.

Together, these results demonstrate that *CLDN2* loss exerts a dual anti‐tumor effect: it intrinsically suppresses NSCLC growth and extrinsically primes cells for cisplatin response. Mechanistically, this chemosensitization is achieved by selectively inhibiting the drug efflux pump MRP2, thereby promoting intracellular cisplatin accumulation and synergistic apoptosis.

### 3.4 *CLDN2* Silencing Synergizes With DDP to Suppress the Progression and Toxicity of NSCLC

**FIGURE 4:**
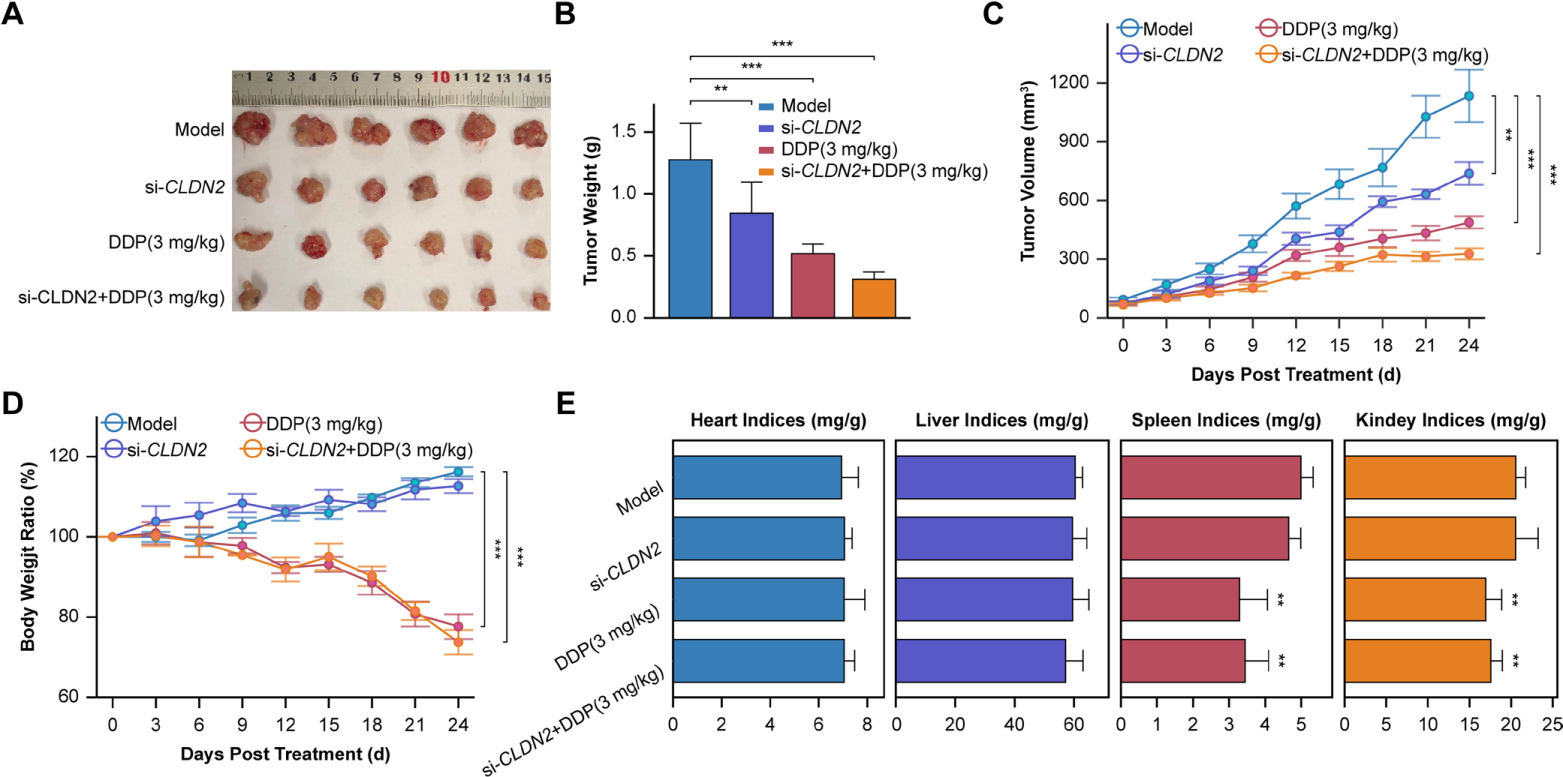
*CLDN2* silencing combined with cisplatin (DDP) modulates tumor growth and systemic toxicity in NSCLC xenograft models. (A) Representative tumor images from Model (untreated), si‐*CLDN2* monotherapy, DDP (3 mg/kg) monotherapy, and combination treatment groups (*n* = 6). (B) Tumor weight quantification across groups. Data are expressed as mean ± SD (*n* = 6). Student's *t*‐test, **p < 0.01, ***p < 0.001. (C) Longitudinal monitoring of tumor volume over time (days post‐treatment). Data are expressed as mean ± SD (*n* = 6). Two‐way ANOVA, **p < 0.01, ***p < 0.001. (D) Body weight ratios relative to baseline during treatment. Data are expressed as mean ± SD (*n* = 6). Two‐way ANOVA, ***p < 0.001. (E) Organ indices (heart, liver, spleen, kidney) normalized to body weight. Data are expressed as mean ± SD (*n* = 6). Student's *t*‐test, **p < 0.01.

*CLDN2* silencing significantly inhibited tumor growth and synergistically enhanced DDP efficacy in NSCLC xenograft models. Tumors in the untreated model group exhibited progressive increases in volume and mass, whereas the si‐*CLDN2* + DDP combination group showed maximal suppression (Figure 4A,B). Longitudinal monitoring delineated divergent tumor dynamics across cohorts, with untreated controls exhibiting sustained growth, si‐*CLDN2* monotherapy inducing partial suppression, DDP monotherapy driving transient stabilization prior to relapse, and the combination cohort achieving sustained regression (Figure 4C).

Systemic toxicity profiles differed significantly across treatment groups. Body weight remained stable in both the Model group and si‐*CLDN2* monotherapy group, whereas DDP monotherapy and combination therapy groups induced progressive weight loss (Figure 4D). Organ index analysis revealed that DDP monotherapy and combination therapy groups exhibited reduced spleen indices (a marker of splenic toxicity) and elevated renal toxicity indices, consistent with the known nephrotoxic effects of DDP (Figure 4E).

This in vivo study confirms that *CLDN2* silencing synergizes with cisplatin to achieve superior and sustained tumor regression in NSCLC. However, this combination retains the characteristic systemic toxicity profile associated with cisplatin monotherapy, indicating that its enhanced therapeutic window stems primarily from increased efficacy rather than reduced toxicity.

### 3.5 *miR‐194‐3p* Suppresses *CLDN2* Through Dual 3′ UTR Binding in NSCLC

**FIGURE 5:**
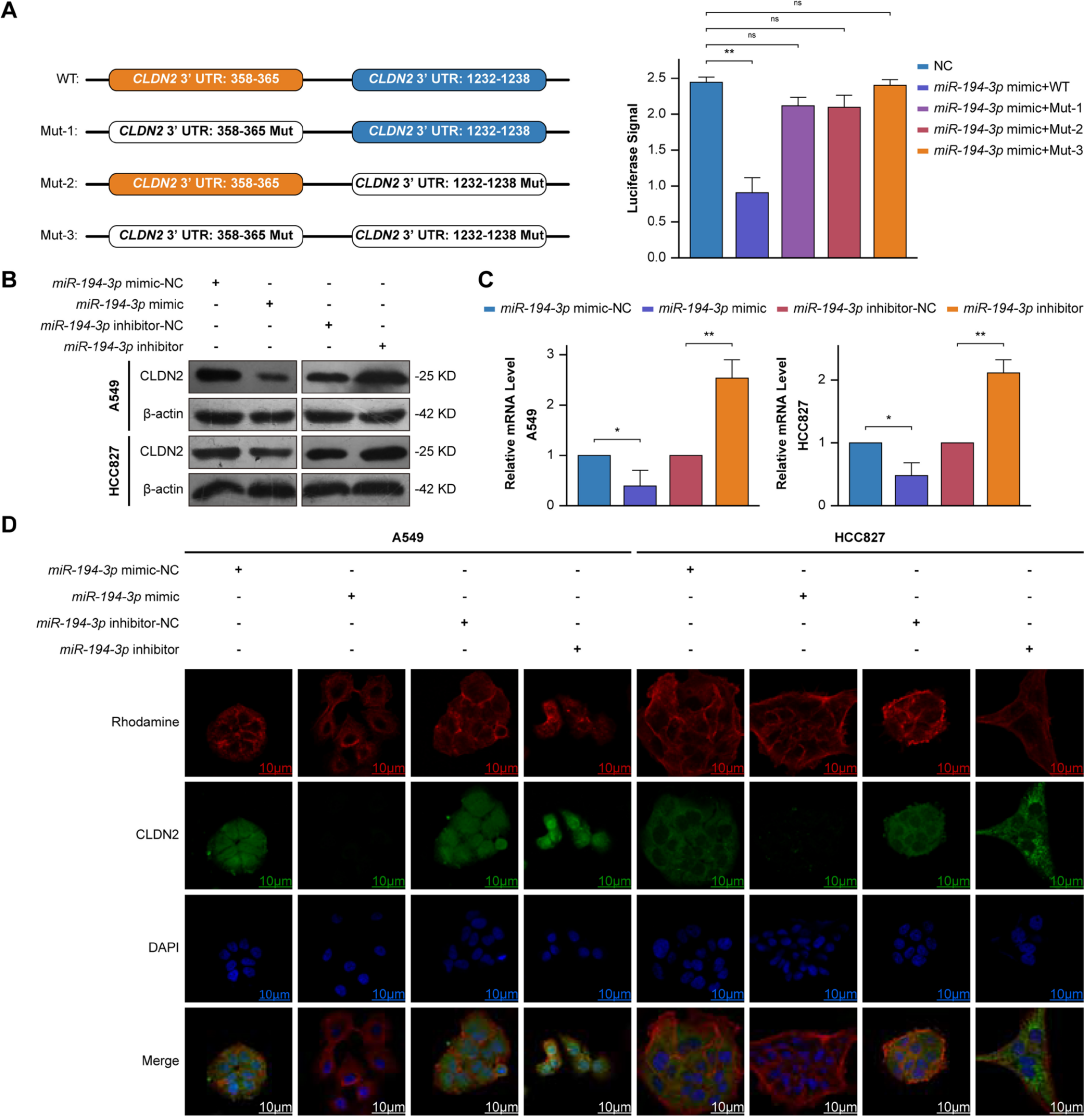
*miR‐194‐3p* regulates *CLDN2* expression and localization via dual 3′ UTR binding in NSCLC. (A) Left: Schematic of *CLDN2* 3′ UTR wild‐type (WT) and mutated (Mut‐1, Mut‐2, Mut‐3) binding sites (358–365 and 1232–1238). Right: Dual‐luciferase reporter assays in 293 T cells co‐transfected with *miR‐194‐3p* mimics and WT/Mut constructs. Data are expressed as mean ± SD (*n* = 3). Student's *t*‐test, **p < 0.01, ns: Not significant. (B) *CLDN2* protein levels in A549 and HCC827 cells transfected with *miR‐194‐3p* mimics‐NC, *miR‐194‐3p* mimics, *miR‐194‐3p* inhibitor‐NC or *miR‐194‐3p* inhibitor (*n* = 3). (C) *CLDN2* mRNA levels across treatment groups. Data are expressed as mean ± SD (*n* = 3). Student's *t*‐test, *p < 0.05, **p < 0.01. (D) Confocal imaging of *CLDN2* (green), Rhodamine membrane marker (red), and nuclei (DAPI, blue) in A549 and HCC827 cells (scale bars: 10 μm, *n* = 3).

Bioinformatic screening (miRDB, TargetScan) identified miR‐194‐3p as a putative regulator of *CLDN2*, with evolutionarily conserved binding motifs at positions 358–365 and 1232–1238 of the *CLDN2* 3′ UTR (Tables 1, 2). In 293 T cells, dual‐luciferase assays demonstrated that miR‐194‐3p overexpression significantly suppressed wild‐type *CLDN2* 3′ UTR reporter activity. Site‐directed mutagenesis at either predicted site (358–365 or 1232–1238) or concurrent mutation of both sites abrogated this suppression, rendering luciferase activity indistinguishable from controls (Figure 5A).

**TABLE 1:** miRDB screening identifies *miR‐194‐3p* as a putative upstream regulator of *CLDN2*.

Target score	miRNA name	Gene symbol	Gene description
86	*hsa‐miR‐194‐3p*	*CLDN2*	claudin 2
85	*hsa‐miR‐6810‐5p*	*CLDN2*	claudin 2
83	*hsa‐miR‐153‐5p*	*CLDN2*	claudin 2

**TABLE 2:** TargetScan mapping reveals *miR‐194‐3p* binding loci in *CLDN2* 3′ UTR.

	Predicted consequential pairing of target region (top) and miRNA (bottom)	Site type	Context++ score	Context++ score percentile
Position 358–365 of *CLDN2* 3′ UTR	5′ACCCACUAAUCACAU**CCCACUHA**	8 mer	−0.42	99
*hsa‐miR‐194‐3p*	3′GUCUAUUGUCGUCG**GGGUGAC**C			
Position 813–819 of *CLDN2* 3′ UTR	5′CAGCGGACUCUGACU**CCACUGA**G	7 mer‐A4	−0.08	75
*hsa‐miR‐194‐3p*	3′GUCUAUUGUCGUCG**GGGUGAC**C			
Position 1232–1238 of *CLDN2* 3′ UTR	5′GCCCCUUCAGAUUCC**CCCACUG**U	7 mer‐m8	−0.20	92
*hsa‐miR‐194‐3p*	3′GUCUAUUGUCGUCG**GGGUGAC**C			

In NSCLC cell lines, transfection of a *miR‐194‐3p* mimic reduced *CLDN2* protein abundance (Figure 5B) and transcript levels (Figure 5C). Confocal microscopy revealed attenuated *CLDN2* membrane localization in mimic‐treated cells, consistent with functional downregulation (Figure 5D). Conversely, transfection of a miR‐194‐3p antisense inhibitor elevated *CLDN2* protein and mRNA expression above baseline levels in both A549 and HCC827 cells (Figure 5B,C). Notably, *CLDN2* membrane signal intensity in inhibitor‐treated cells exceeded that of untreated controls, indicating ectopic overexpression rather than restoration of physiological expression levels (Figure 5D). Notably, given the critical regulatory role of *miR‐194‐3p* on CLDN2 established above, we further investigated the effect of Art B on miR‐194‐3p expression in NSCLC cells. Our results demonstrate that Art B significantly upregulates the intracellular level of *miR‐194‐3p* (Figure S1).

This study identifies *miR‐194‐3p* as a key post‐transcriptional regulator of *CLDN2* in NSCLC, directly suppressing its expression via two evolutionarily conserved binding sites in its 3′ UTR. The additional finding that Art B upregulates *miR‐194‐3p* suggests this microRNA as a potential upstream mediator of Art B's pharmacological action.

### 3.6 *miR‐194‐3p* Enhances Cisplatin Efficacy Through MRP2 Silencing in NSCLC

Overexpression of *miR‐194‐3p* suppressed NSCLC cell proliferation and potentiated DDP sensitivity. Longitudinal monitoring revealed sustained growth arrest over 96 h in A549 and HCC827 cells following *miR‐194‐3p* overexpression, whereas inhibition of endogenous *miR‐194‐3p* partially rescued proliferative capacity (Figure S2A). Morphological assessment demonstrated reduced cellular density and cytoplasmic condensation in miR‐194‐3p‐overexpressing cells, contrasting with maintained structural integrity in inhibitor‐treated groups (Figure S2B).

**FIGURE 6:**
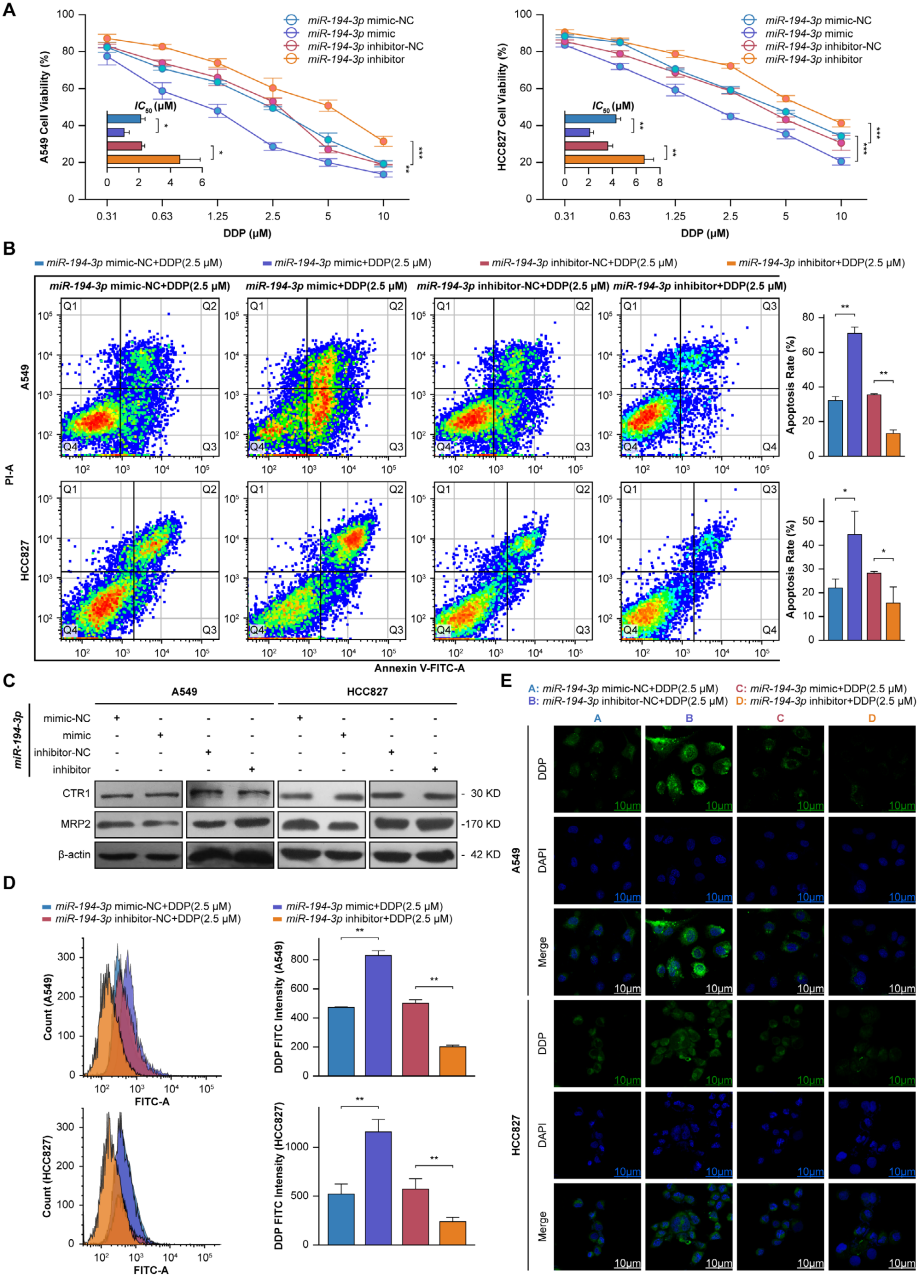
*miR‐194‐3p* modulates DDP sensitivity and apoptotic responses in NSCLC models. (A) Left: A549 and Right: HCC827 cell viability under escalating DDP concentrations (0.31–10 μM) in *miR‐194‐3p* minic‐NC, *miR‐194‐3p* mimic, *miR‐194‐3p* inhibitor‐NC, and *miR‐194‐3p* inhibitor. The histogram displays the *IC*_50_ values corresponding to each curve. Data are expressed as mean ± SD (*n* = 3). Cell viability curves using curves two‐way ANOVA, **p < 0.01, ***p < 0.001; histogram using Student's *t*‐test, *p < 0.05, **p < 0.01. (B) Left: Annexin V/PI flow cytometry plots (quadrants Q1–Q4: Necrosis/viable/early/late apoptosis). Right: Apoptotic rate quantification. Data are expressed as mean ± SD (*n* = 3). Student's *t*‐test, *p < 0.05, **p < 0.01. (C) Western blot analysis of MRP2 and CTR1 expression (*n* = 3). (D) Left: Cell counts normalized to controls; Right: Fluorescence intensity of intracellular DDP (2.5 μM) retention. Data are expressed as mean ± SD (*n* = 3). Student's *t*‐test, **p < 0.01. (E) Confocal imaging of DDP (green) and nuclei (DAPI, blue) under *miR‐194‐3p* mimic/inhibitor+DDP (2.5 μM) treatments. (scale bars: 10 μm, *n* = 3).

Dose–response cytotoxicity assays demonstrated that *miR‐194‐3p* overexpression enhanced DDP‐induced cell death across increasing concentrations, while inhibition of *miR‐194‐3p* attenuated chemosensitization (Figure 6A). Apoptotic profiling confirmed significantly increased cell death in miR‐194‐3p‐overexpressing groups, characterized by elevated proportions of cells in early and late apoptotic phases; this phenotype was reversed by *miR‐194‐3p* inhibitor co‐treatment (Figure 6B).

Mechanistically, *miR‐194‐3p* mediated post‐transcriptional repression of MRP2 (a canonical multidrug resistance efflux transporter) without affecting CTR1 expression (Figure 6C). This MRP2 downregulation correlated with increased intracellular DDP retention, evidenced by enhanced platinum‐DNA adduct formation in flow cytometric analysis (Figure 6D) and perinuclear DDP compartmentalization in confocal microscopy (Figure 6E).

These findings elucidate a functional role for *miR‐194‐3p* in enhancing cisplatin efficacy, mediated through its post‐transcriptional repression of the efflux transporter MRP2. This leads to increased intracellular drug accumulation and sensitizes NSCLC cells to apoptosis, positioning *miR‐194‐3p* as a key modulator of chemotherapeutic response.

### 3.7 The miR‐194‐3p/CLDN2 Axis Modulates DDP Sensitivity Through MRP2 in NSCLC

Combined *CLDN2 silencing* and miR‐194‐3p inhibition rescued proliferative capacity after *CLDN2* depletion. Longitudinal monitoring showed restored growth kinetics in A549 and HCC827 cells over 96 h following dual intervention, contrasting with sustained suppression in CLDN2‐silenced groups (Figure S2C). Morphological analysis revealed preserved cell–cell adhesion and attenuated cytoplasmic condensation in co‐treated cells, whereas *CLDN2* knockdown alone induced disrupted cellular architecture (Figure S2D).

**FIGURE 7:**
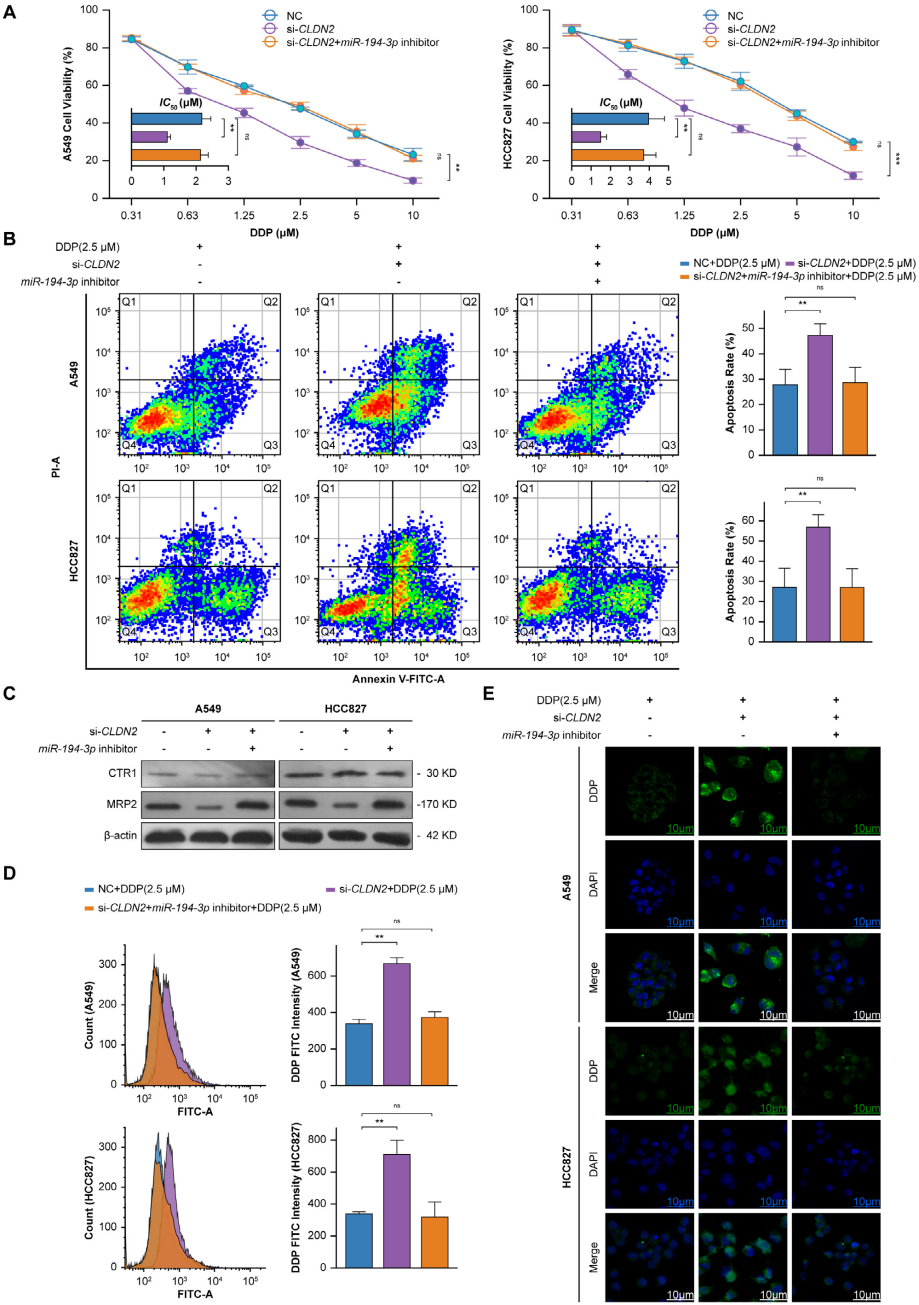
*CLDN2* silencing combined with *miR‐194‐3p* inhibition modulates DDP‐induced cellular responses in NSCLC. (A) Left: A549 and Right: HCC827 cell viability under escalating DDP concentrations (0.31–10 μM). Groups: NC, si‐*CLDN2*, and si‐*CLDN2* + *miR‐194‐3p* inhibitor. The histogram displays the *IC*_50_ values corresponding to each curve. Data are expressed as mean ± SD (*n* = 3). Cell viability curves using curves two‐way ANOVA, **p < 0.01, ***p < 0.001, ns: Not significant; histogram using Student's *t*‐test, **p < 0.01, ns: Not significant. (B) Left: Annexin V‐FITC/PI flow cytometry plots. Right: Quantified apoptotic rates across groups. Data are expressed as mean ± SD (*n* = 3). Student's *t*‐test, **p < 0.01, ns: Not significant. (C) Western blot analysis of CTR1 and MRP2 expression (*n* = 3). (D) Left: FITC‐A fluorescence intensity (arbitrary units) reflecting DDP (2.5 μM) levels. Right: Representative flow cytometry histograms. Data are expressed as mean ± SD (*n* = 3). Student's *t*‐test, **p < 0.01, ns: Not significant. (E) Confocal imaging of DDP (green) and DAPI (blue) in NC + DDP (2.5 μM), si‐*CLDN2* + DDP (2.5 μM), and si‐*CLDN2* + *miR‐194‐3p* inhibitor groups+DDP (2.5 μM). (scale bars: 10 μm, *n* = 3).

*CLDN2* silencing significantly enhanced DDP‐induced cytotoxicity, as shown by viability across increasing drug concentrations (Figure 7A). This hypersensitivity was partially rescued by miR‐194‐3p inhibition, restoring baseline chemoresistance. Apoptosis assays corroborated heightened cell death in *CLDN2*‐silenced groups, with expanded early and late apoptotic populations, whereas miR‐194‐3p inhibition attenuated this phenotype (Figure 7B). Mechanistically, miR‐194‐3p blockade abrogated *CLDN2* knockdown‐mediated MRP2 suppression, restoring MRP2 to near‐physiological levels (Figure 7C).

This MRP2 recovery correlated with decreased intracellular DDP accumulation, quantified by reduced platinum‐DNA adduct formation (Figure 7D). Confocal imaging demonstrated distinct spatial redistribution patterns across cohorts, with *CLDN2*‐silenced cells exhibiting perinuclear DDP aggregation co‐localizing with nuclear DNA, whereas co‐treatment with miR‐194‐3p inhibitor showed diffuse cytoplasmic DDP distribution in vesicular compartments (Figure 7E). These spatial patterns aligned with MRP2's efflux function—perinuclear accumulation indicates impaired export, while cytoplasmic dispersion reflects restored transporter activity.

Our results position *miR‐194‐3p* as the principal mediator through which *CLDN2* silencing enhances cisplatin sensitivity. Inhibition of *miR‐194‐3p* reverses the chemosensitizing effects of *CLDN2* depletion by restoring MRP2 expression and its efflux function, thereby altering the spatial distribution and intracellular accumulation of cisplatin.

## 4 Discussion

Artemisinin derivatives hold significant therapeutic potential, demonstrating tumor‐selective cytotoxicity and favorable pharmacodynamic properties in preclinical evaluations [7]. Art B specifically shows robust antitumor efficacy in hepatocellular carcinoma, leukemia, and gastric cancer models, yet its precise molecular targets within NSCLC remain uncharted [27, 28]. Previous work by our group confirmed Art B's capacity to inhibit NSCLC proliferation, metastatic dissemination, and cisplatin chemoresistance; nevertheless, the mechanistic foundation underpinning these phenotypes was unresolved. Here, we delineate the miR‐194‐3p/CLDN2 axis as the primary effector pathway mediating Art B's anticancer activity. This discovery not only resolves a key uncertainty regarding Art B's mode of action but also establishes a novel molecular blueprint for overcoming chemoresistance in NSCLC treatment, moving beyond phenomenological observation to mechanistic understanding.

As a core constituent of leaky epithelial tight junctions, *CLDN2* governs paracellular cation/water flux—a physiological function hijacked in malignancies to accelerate tumorigenesis [29]. Pathological *CLDN2* overexpression defines aggressiveness in gastric, colorectal, lung, breast, renal carcinomas, and osteosarcoma, correlating with dismal prognosis [26, 30, 31]. In lung adenocarcinoma specifically, *CLDN2* elevation versus normal bronchial epithelium drives oncogenesis; its ablation impedes proliferation, metastasis and chemoresistance in preclinical systems [17, 18, 32, 33]. Epigenetic modulators and flavonoids attenuate NSCLC progression by suppressing *CLDN2*, with phenotypic rescue upon *CLDN2* restoration confirming its centrality [19, 34, 35]. Notably, ECL2‐mimetic peptides induce *CLDN2* internalization and lysosomal degradation, provoking necrotic death—a promising therapeutic strategy [36]. Our chemoproteomic studies, validated via TCGA analysis and functional rescue experiments, establish that *CLDN2* orchestrates cisplatin resistance through MRP2‐dependent efflux pumps, depleting intracellular drug accumulation. This finding is significant because it elucidates a non‐canonical, transporter‐mediated resistance mechanism for *CLDN2*, positioning it as a linchpin of chemoresistance and a compelling druggable target whose inhibition could prevent drug efflux.

*CLDN2* expression is dynamically regulated by microRNAs (miRNAs), post‐transcriptional modulators that fine‐tune oncogenic networks. Established miRNAs including miR‐488, miR‐16, and the miR‐199a‐5p/214‐3p cluster engage complementary sequences within the *CLDN2* 3′ UTR to inhibit translation [19, 37]. We identify miR‐194‐3p as a previously uncharacterized *CLDN2* repressor targeting dual loci (nt 358–365 and 1232–1238) in its 3′ UTR. This miRNA functions as a tumor suppressor in lung adenocarcinoma by impeding SLC12A5/TWIST1‐driven metastasis and in nasopharyngeal carcinoma by counteracting PTPRG‐AS1‐mediated invasion [38, 39]. Pharmacological *miR‐194‐3p* induction via CG200745 inhibits cholangiocarcinoma growth, while its restoration suppresses breast cancer progression across aggressive subtypes [40, 41, 42]. Crucially, we demonstrate that *miR‐194‐3p* directly suppresses *CLDN2* in NSCLC at both transcriptional and translational levels, reversing proliferative capacity and cisplatin resistance—effects potentiated by Art B through specific *miR‐194‐3p* upregulation. This mechanistic linkage positions the miR‐194‐3p/CLDN2 axis as a druggable epigenetic switch governing chemoresistance. Although preclinical artemisinin data suggest favorable toxicity profiles, Art B's long‐term safety—particularly in cisplatin combination regimens—warrants rigorous clinical evaluation. The translational implication of our work lies in the potential of targeting the miR‐194‐3p/CLDN2 axis, either directly with Art B or through future miRNA‐based therapeutics, to resensitize resistant NSCLC tumors.

## 5 Conclusion

This work delineates *CLDN2* as a molecular driver of NSCLC chemoresistance through integrated functional genomics and experimental validation, establishing its non‐canonical resistance mechanism via MRP2‐mediated cisplatin extrusion. We elucidate the miR‐194‐3p/CLDN2 axis as a master regulatory circuit governing malignancy and nominate artemisinin derivative Art B as its first‐in‐class modulator that enhances cisplatin cytotoxicity through specific *miR‐194‐3p* activation. These findings resolve persistent ambiguities regarding artemisinin's antitumor efficacy while positioning *CLDN2* as a druggable target. Current conclusions, however, rely predominantly on in vitro models and lack validation in histological subtype‐specific patient‐derived systems. Future studies must prioritize translational development of Art B‐cisplatin combinations to counter evolved resistance, alongside advancing CLDN2‐targeted biologics or miRNA therapeutics. Critical implementation barriers remain: engineering tissue‐selective delivery platforms for *miR‐194‐3p* mimics and establishing predictive biomarkers for patient stratification.

## Author Contributions

**Ting‐Sha He:** conceptualization (equal), formal analysis (lead), writing – original draft (lead). **Rong‐Hui Chen:** methodology (equal), investigation (equal), data curation (equal). **Jing Feng:** methodology (equal), validation (equal), writing – review and editing (supporting). **Qiang Zhang:** software (equal), formal analysis (supporting). **Xin‐Ling Li:** investigation (equal), resources (supporting). **Jia‐Hui Han:** visualization (equal), project administration (supporting). **Tian‐Ze Chen:** resources (equal), supervision (supporting). **Rong‐Min Yu:** validation (equal), writing – review and editing (equal), supervision (equal). **Li‐Yan Song:** conceptualization (equal), funding acquisition (lead), supervision (lead), writing – review and editing (equal). **Wei‐Juan Huang:** conceptualization (lead), funding acquisition (equal), project administration (lead), writing – review and editing (equal).

## Funding

This work was supported by grants from the National Natural Science Foundation of China (82574486 and 82003774 to W.‐J Huang; 82174019 and 81673646 to L.‐Y Song), and Guangdong Provincial Natural Science Foundation (2023A1515012, and 2024A1515010185 to W.‐J Huang).

## Ethics Statement

The mice used in this study were purchased from Beijing Huafukang Company and housed in the Experimental Animal Center of Jinan University. After a ten‐day quarantine period, the experiment was conducted. This study was reviewed and approved by the Animal Ethics Committee of Jinan University (Approval number: IACUC20201125‐06), following the principles of animal welfare and ethics.

## Conflicts of Interest

These authors declare no conflicts of interest.

## Supporting information


**Figure S1:** Bar graphs show the relative expression levels of *miR‐194‐3p* in A549 and HCC827 cells after treatment with negative control (NC) or Art B (6.25 μM). Data are expressed as mean ± SD (*n* = 3). Student's *t*‐test, **p < 0.01.

**Figure S2:** Time‐dependent viability and morphological changes under indicated treatments. (A) Viability curves of *miR‐194‐3p* mimic‐NC, *miR‐194‐3p* mimic, *miR‐194‐3p* inhibitor‐NC, and *miR‐194‐3p* inhibitor groups over 96 h. A549 is shown as circles, and HCC827 as triangles. Data are expressed as mean ± SD (*n* = 3). Two‐way ANOVA, ***p < 0.001. (B) Morphological assessment of *miR‐194‐3p* mimic‐NC, *miR‐194‐3p* mimic, *miR‐194‐3p* inhibitor‐NC, and *miR‐194‐3p* inhibitor‐treated cells. (C) Viability curves of NC, si‐*CLDN2*, and si‐*CLDN2* + *miR‐194‐3p* inhibitor groups. A549 is shown as circles, and HCC827 as triangles. Data are expressed as mean ± SD (*n* = 3). Two‐way ANOVA, ***p < 0.001, ns: not significant. (D) Morphological changes in NC, si‐*CLDN2*, and si‐*CLDN2* + *miR‐194‐3p* inhibitor groups.

**Table S1:** Primer sequences for qPCR amplification of *CLDN2* and *GAPDH*.

## Data Availability

The data that support the findings of this study are available from the corresponding author upon reasonable request.
