# Supplementary figures and images for: Arteannuin B Inhibits NSCLC Cells via Regulating miR‐194‐3p/ CLDN2 Axis
Source: Cancer Med. 2026 Apr 14;15(4):e71796. doi: 10.1002/cam4.71796 (PMC13079067; doi:10.1002/cam4.71796)

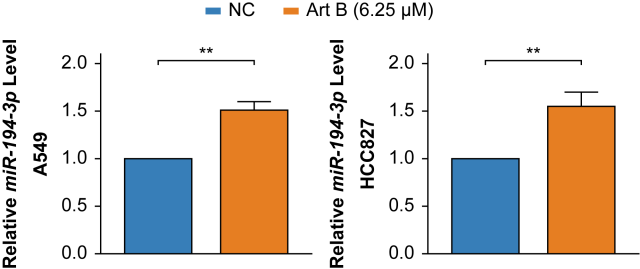

**Figure. Supplementary 1**

Supplement: Supplementary file 1 — Figure S1: Bar graphs show the relative expression levels of miR‐194‐3p in A549 and HCC827 cells after treatment with negative control (NC) or Art B (6.25 μM). Data are expressed as mean ± SD (n = 3). Student's t‐test, **p < 0.01. [file CAM4-15-e71796-s003.pdf]

**A**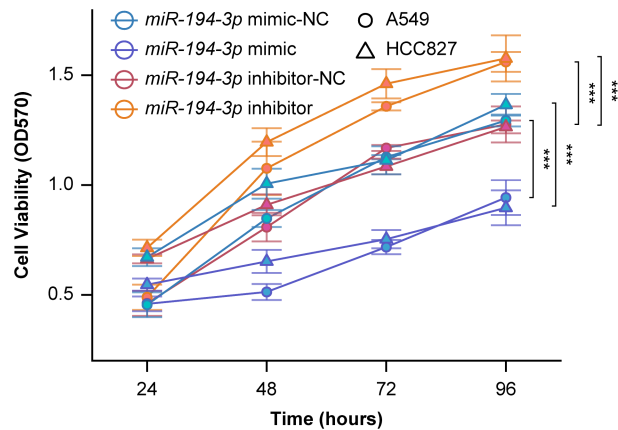**B**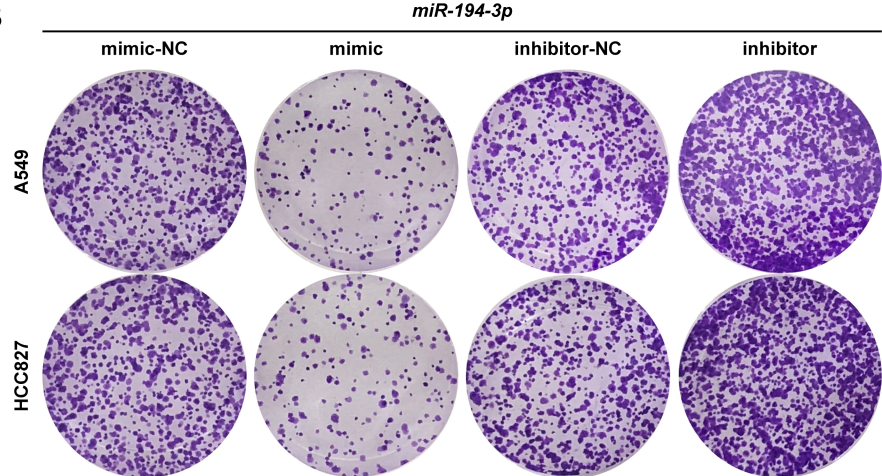**C**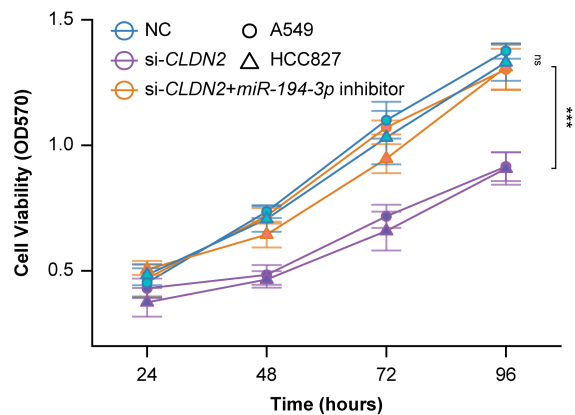**D**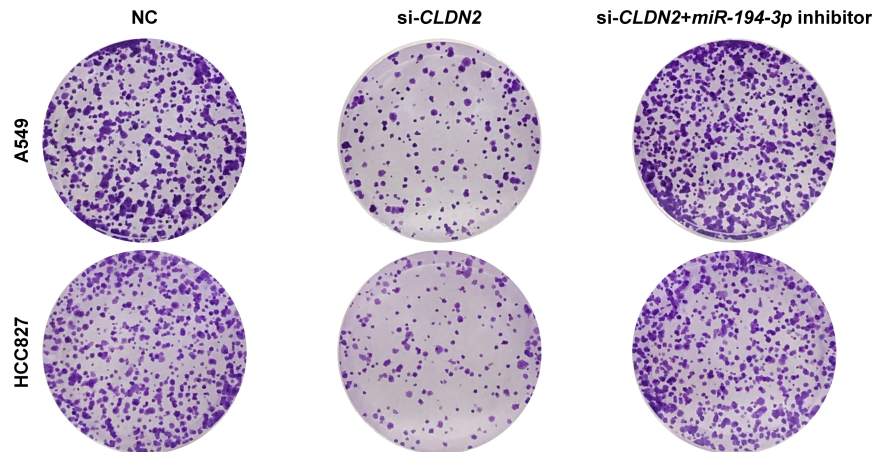

**Figure. Supplementary 2**

Supplement: Supplementary file 2 — Figure S2: Time‐dependent viability and morphological changes under indicated treatments. (A) Viability curves of miR‐194‐3p mimic‐NC, miR‐194‐3p mimic, miR‐194‐3p inhibitor‐NC, and miR‐194‐3p inhibitor groups over 96 h. A549 is shown as circles, and HCC827 as triangles. Data are expressed as mean ± SD (n = 3). Two‐way ANOVA, ***p < 0.001. (B) Morphological assessment of miR‐194‐3p mimic‐NC, miR‐194‐3p mimic, miR‐194‐3p inhibitor‐NC, and miR‐194‐3p inhibitor‐treated cells. (C) Viability curves of NC, si‐CLDN2, and si‐CLDN2 + miR‐194‐3p inhibitor groups. A549 is shown as circles, and HCC827 as triangles. Data are expressed as mean ± SD (n = 3). Two‐way ANOVA, ***p < 0.001, ns: not significant. (D) Morphological changes in NC, si‐CLDN2, and si‐CLDN2 + miR‐194‐3p inhibitor groups. [file CAM4-15-e71796-s002.pdf]
