# Supplementary material for: Arteannuin B Inhibits NSCLC Cells via Regulating miR‐194‐3p/ CLDN2 Axis
Source: Cancer Med. 2026 Apr 14;15(4):e71796. doi: 10.1002/cam4.71796 (PMC13079067; doi:10.1002/cam4.71796)
Supplement: Supplementary file 3 — Table S1: Primer sequences for qPCR amplification of CLDN2 and GAPDH. [file CAM4-15-e71796-s001.docx]

**TABLE Supplementary |** Primer sequences for qPCR amplification of *CLDN2* and *GAPDH*.

| **Primers** | **Sequence** | |
| --- | --- | --- |
|  | Forward（5’-3’） | Reverse（5’-3’） |
| CLDN2 | ATTGTGACAGCAGTTGGCTT | CTATAGATGTCACACTGGGTGATG |
| GAPDH | ACCACAGTCCATGCCATCAC | TCCACCACCCTGTTGCTGTA |
